# Routine viral load monitoring in HIV‐infected infants and children in low‐ and middle‐income countries: challenges and opportunities

**DOI:** 10.1002/jia2.25001

**Published:** 2017-11-24

**Authors:** Stephen M Arpadi, Stephanie Shiau, Eduarda Pimentel De Gusmao, Avy Violari

**Affiliations:** ^1^ ICAP Columbia University New York NY USA; ^2^ Department of Pediatrics College of Physicians & Surgeons Columbia University New York NY USA; ^3^ Department of Epidemiology Mailman School of Public Health Columbia University New York NY USA; ^4^ Empilweni Services and Research Unit Department of Paediatrics and Child Health Faculty of Health Sciences Rahima Moosa Mother and Child Hospital University of the Witwatersrand Johannesburg South Africa; ^5^ Perinatal Health Research Unit University of Witwatersrand Johannesburg South Africa

**Keywords:** viral load, HIV/AIDS, implementation, paediatrics, children, monitoring

## Abstract

**Introduction:**

The objective of this commentary is to review considerations for implementing routine viral load (VL) monitoring programmes for HIV‐infected infants and children living in low‐ and middle‐income countries (LMIC). Since 2013, the World Health Organization (WHO) guidelines recommend VL testing as the preferred monitoring approach for all individuals treated with ART in order to assess treatment response, detect treatment failure and determine the need to switch to a second‐line regimen in a timely manner. More recently, WHO guidelines from 2016 identify HIV‐infected infants and children as a priority group for routine VL monitoring.

**Discussion:**

There are a number of reasons why HIV‐infected infants and children should be prioritized for routine VL monitoring. Data from national VL monitoring programmes as well as systematic reviews and meta‐analyses from LMIC indicate rates of viral suppression are lower for infants and children compared to adults. The number of antiretroviral drugs and palatable formulations suitable for young children are limited. In addition, emotional and developmental issues particular to children can make daily medication administration difficult and pose a challenge to adherence and achievement of sustained viral suppression. VL monitoring can be instrumental for identifying those in need of additional adherence support, reducing regimen switches and preserving treatment options. The needs of infants and children warrant consideration in all aspects of VL monitoring services. If capacity for paediatric venipuncture is not assured, platforms that accept dried blood spot specimens are necessary in order for infants and children to have equitable access. Healthcare systems also need to prepare to manage the substantial number of infants and children identified with elevated VL, including adherence interventions that are appropriate for children. Establishing robust systems to evaluate processes and outcomes of routine VL monitoring services and to support drug forecasting and supply management is essential to determine best practices for infants and children in LMIC.

**Conclusions:**

The particular concerns of HIV‐infected infants and children warrant attention during all phases of planning and implementation of VL monitoring services. There are a number of key areas, including frequency of monitoring, blood specimen type and adherence challenges, where specific approaches tailored for infants and children may be required.

## Introduction

Access to antiretroviral therapy (ART) in children <15 years has greatly expanded, with an estimated 920,000 children <15 years reportedly receiving ART in 2016 compared to 18,000 children in 2000 1. The new treatment paradigm recommends initiating ART at earlier ages in all children independent of CD4 T‐cell counts or clinical stage. This approach could expedite access to treatment for an additional 1,180,000 HIV‐infected children in need of ART. Developing and implementing monitoring strategies to optimize outcomes in children on ART in low‐ and middle‐income countries (LMIC), where over 90% of children with HIV live, is a critical clinical and public health challenge.

Since 2013, the World Health Organization (WHO) guidelines recommend viral load (VL) testing as the preferred monitoring approach for all individuals treated with ART in order to assess treatment response, detect treatment failure and determine the need to switch to a second‐line regimen in a timely manner 2. More recently, WHO guidelines from 2016 identify HIV‐infected infants and children as a priority group for preferential routine VL monitoring 2. The objective of this commentary is to review considerations for implementing routine VL monitoring for HIV‐infected infants and children in LMIC.

### Reasons for prioritizing HIV‐infected infants and children for VL monitoring services

HIV‐infected infants and children are considered a priority group for routine VL monitoring for a number of reasons.

The efficacy of early ART for achieving viral suppression, promoting immune reconstitution, and reducing morbidity and mortality in children is well‐established 3, 4. However, until recently, data on rates of VL suppression among children undergoing routine monitoring in LMICs in contrast to more targeted VL testing of children suspected of treatment failure were unavailable. Initial results from national routine VL monitoring programmes in Kenya and Uganda that include large representative samples with age‐disaggregated reporting indicate that rates of viral suppression are low for infants, children and adolescents compared to adults 5, 6. The overall rate of viral suppression among children in five eastern–southern African countries with nationally representative data from routine viral load monitoring was 62% 7. Similar low rates of viral suppression are reported from earlier studies from single or multiple facilities in LMIC 8, 9. Lower rates of viral suppression among paediatric and adolescent patients compared to adults have also been reported in several systematic reviews and meta‐analyses. A meta‐analysis by Ciaranello *et al*. 10 in 2009 using data from nine studies in resource‐limited settings collected from 1997 to 2008 found the pooled estimate for 12‐month viral suppression (HIV RNA <400 copies/mL) in children <15 years to be 70% (95% confidence interval [CI]: 67–73). A large meta‐analysis conducted in 2016 of both observational studies and randomized controlled trials evaluating viral suppression identified 72 studies reporting on 51,374 children <18 years. After 12 months on first‐line ART, viral suppression was achieved by 64.7% (95% CI: 57.5–71.8) in studies conducted from 2000 to 2005, 74.2% (95% CI: 70.2–78.2) in studies conducted from 2006 to 2009 and 72.7% (95% CI: 62.6–82.8) in studies conducted after 2010 11. These rates are considerably lower than those typically observed in adults, including in a meta‐analysis of virologic outcomes in adults, which found viral suppression rates >80% in the first five years on ART 12.

The number of antiretroviral drugs and palatable formulations suitable for young children are limited 2, making avoidance of unnecessary changes in ART particularly important. For example, nevirapine and efavirenz, first‐generation non‐nucleoside reverse transcriptase inhibitors (NNRTI), are not recommended for children less than three years of age 2. This is due to findings from clinical trials that demonstrated elevated rates of failure among children on NNRTI‐based regimens compared to protease inhibitor (PI)‐based regimens regardless of prior exposure to NNRTI for prevention of mother‐to‐child transmission (PMTCT) 13, 14. Thus, these agents, which for many years have been the cornerstone of first‐line ART for adults living in LMIC, are not the preferred option for children under three years for whom ritonavir‐boosted lopinavir (LPV/r)‐based regimens are used as first‐line ART. In addition, for a number of reasons including cold chain limitations, LPV/r may not be consistently available in all settings. Newer agents, such as darunavir, etravirine and raltegravir, used in second‐line regimens are difficult to acquire for children failing first‐line PI‐regimens in many LMIC, and are often only available through donation programmes if at all. Routine regularly scheduled VL monitoring has the potential to preserve treatment options through early identification of those with non‐suppression who might benefit from timely intensified adherence support to prevent treatment failure and the need for regimen changes.

Finally, there are issues particular to children that may undermine ART adherence and contribute to poorer virologic outcomes. Due to a number of emotional and developmental factors, daily medication administration to infants and young children can be extremely difficult, especially with bad tasting preparations, and child‐caregiver conflicts over medication are not uncommon 15, 16. Swallowing of tablets, when available in paediatric formulations can also be difficult for many children. A child's adherence is also vulnerable to changes in social environments. As children are reliant on adult caretakers for monitoring home supply and administration of ART and clinic visits, caretaker changes or alterations in household routines are a frequent cause of disruptions in adherence 17. In addition, dose‐adjusting is required to account for growth and failure to do so may result in under‐dosing of one or more antiretroviral agents in a regimen.

## Discussion

**Figure 1:**
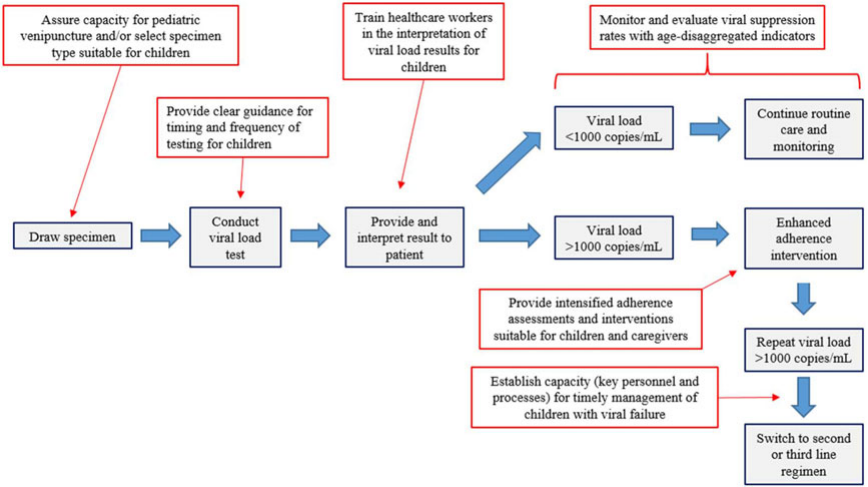
Aspects along the viral load cascade requiring special consideration for children in the planning and implementation of a viral load monitoring programme.

The particular needs of infants and young children should be considered at each phase of planning and implementation of VL monitoring at all levels of the healthcare system, ranging from national programmes to individual health facilities. In this section, we discuss key aspects in implementing VL monitoring programmes, where attention to the needs of infants and children is warranted. An overview of these aspects is provided in Figure 1.

Planning and implementation of routine VL monitoring services would benefit from considering lessons learned from national early infant diagnosis (EID) programmes or evaluating existing programmes that monitor VL testing for children suspected of treatment failure. A number of countries report shortcomings, including inadequate specimen collection and transport systems, inefficient (e.g. duplicative) lab information systems, test kit stock outs, insufficient technical personnel, long turnaround times, inefficient reporting of results and suboptimal clinical decision‐making once results are returned to clinical care sites 18, 19, 20, 21. In some environments, less than half of EID results were ever available for patient care decisions 22.

National decision‐making and planning bodies (e.g. technical working groups) should include individuals with technical expertise relevant to paediatrics. Updated national guidelines should include VL monitoring recommendations specific to infants and children. If a phase‐in approach for implementation is planned, priority populations for early access should include infants and children.

The optimal timing and frequency for routine VL monitoring in infants and children on ART has not been established, and currently, there is little evidence to inform this question. Nonetheless, it is essential that clear guidance be provided even if considered provisional until additional studies are available. The WHO advises VL monitoring at 6, 12 months and then every 12 months for patients that are stable on first‐line ART and grades the supportive evidence for this recommendation as very low quality 24. A number of countries with national VL monitoring programmes currently endorse the WHO recommendation of a single schedule for VL monitoring for non‐pregnant and breastfeeding ART‐treated adults, as well as for infants and children 23, 24, 25, while others have adopted paediatric specific schedules. For example, the Botswana National Guidelines recommend VL testing for infants and children on ART every three months 26. Evaluating whether more frequent monitoring together with support for adherence leads to lower rates of first‐line treatment failure is an important area of future research.

It is also important to consider infants and children when selecting specimen type and platform or assay for VL testing. Apart from specialized paediatric care settings, reliance on plasma‐based specimens poses a major obstacle to implementation of VL monitoring for infants and children 27. Unless or until capacity for paediatric venipuncture is assured, the only practical way for young children and infants on ART to access VL monitoring is by means of assay platforms that accept dried blood spot (DBS) specimens, a number of which have been validated against plasma 28, 29. Introduction of point‐of‐care (POC) VL platforms may also provide advantages, particularly for children in situations where turnaround times for results from central labs may undermine the value of monitoring schedules that call for shorter testing intervals.

There are also specific training issues for healthcare workers relevant to VL monitoring for infants and children. The currently recommended criteria for viral failure (i.e. persistent VL above 1000 copies/mL after at least six months of taking ART) by WHO is the same for all ages 2. Here, again the recommendation for the optimal threshold to define viral failure and criteria for switching ART is provisional and may require adjustments as additional evidence becomes available. The threshold of 1000 copies/mL is based on evidence mainly from studies conducted in adults suggesting that risk of HIV disease progression is very low below this threshold 30, as well as evidence that intermittent low‐level viraemia (50–1000 copies/mL) is not associated with short term treatment failure 31. Results from a randomized clinical trial conducted among children ages 0.1–17.8 years (median 6.5 years) starting first‐line therapy found no difference in four‐year VL outcomes when ≥1000 copies/mL was used as the switching threshold compared to ≥30,000 copies/mL 32. However, the higher switching threshold, affects drug‐related resistance, among those on an NNRTI‐based regimen; more nucleoside reverse transcriptase inhibitor (NRTI) mutations were detected in those switching at 30,000 copies compared to those switching at 1000 copies/mL. No differences in clinically important PI or NRTI mutations were detected between the two switching thresholds. The long‐term clinical and virologic outcomes in children when using a threshold of 1000 copies/mL has not yet been evaluated and is an important research question.

In addition, due to high levels of viral replication during the first few months of life, some infants may require more than six months to achieve initial suppression to below 1000 copies/mL 33. Further research is required to determine if obtaining pre‐treatment baseline VL for young infants is warranted in order to assist with the interpretation of VL results on ART. These considerations may become more important with greater emphasis on early ART initiation 3, 4. Healthcare workers will require ongoing training on these issues.

Widespread availability of VL monitoring provides the opportunity for earlier detection of treatment failure and allows for timely switching of ART regimens, as well as avoids unnecessary changes in medications when compared to reliance on CD4 and clinical status alone 34, 35. In addition, detection of elevated VL identifies individuals who might benefit from targeted adherence interventions in order to achieve (re)‐suppression and preserve future treatment options. Healthcare workers and healthcare systems need to prepare and develop capacity to manage the potentially substantial number of infants and children with elevated VL. This entails provision of intensified adherence assessments and interventions that are appropriate for children at various stages of development, as well as for household members and individuals involved in the care of the child 36. Disclosure to the child of their HIV status can also be an important aspect of adherence counselling. There remains a great need to determine the best practices for improving adherence among HIV‐infected children in LMIC, as much of limited prior research was conducted in high income countries 37, 38. Support for effectiveness of a number of adherence interventions in children on viral suppression is available including use of peer‐support, adherence counsellors, educational session and home visits. A review by Bonner *et al*. 39 reported a pooled estimate of 70.5% (95% CI: 56.6–84.4) of repeat VL below 1000 copies/mL found by routine VL testing with prior VL >1000 copies/mL. A smaller study of children by Jobanputra et al. conducted in Swaziland reported that 61% of those with elevated VL who had undergone enhanced adherence counselling had a VL <1000 copies/mL when repeated at least 60 days later 40. Healthcare workers must be knowledgeable about common adherence barriers experienced by infants and children and their caregivers and potential remedies.

Routine VL monitoring can be also anticipated to bring a new urgency to securing or establishing the capacity for timely switches in ART regimens for children with viral failure as demonstrated by persistently elevated VL despite good adherence. As shown in prior studies, VL monitoring is associated with higher rates of second‐line ART 41, 42. Standardized procedures suitable to the context for establishing processes, roles and responsibilities of key persons for switching infants and children to second‐line and third‐line ART regimen are required. An assessment of the human resources and specialized skills of the key cadre(s) for these tasks may be required. In some LMIC, nurses are among the most important prescribers of first‐line ART 43. Future options may include expanding their scope of practice to include switching children to second or third‐line regimens, or establishing other centralized processes as available resources allow.

Finally, monitoring systems that support accurate and timely evaluation of all facets of routine VL monitoring for infants and children are required, including supporting a dynamic drug forecasting, procurement and distribution system that can rapidly respond to change in demand for therapeutic agents required for second‐ and third‐line paediatric ART regimens. Monitoring systems to support the integration of VL data between health facilities and laboratories and between healthcare workers and patients will need to be adapted for paediatric purposes. Quality assessment and improvement activities will depend on the timely availability of age‐disaggregated reports.

## Conclusions

In order for national VL monitoring programmes in LMIC to have a maximal impact on outcomes for all patient groups, the particular concerns of HIV‐infected infants and children warrant attention during all phases of planning and implementation. There are a number of key areas, including frequency of monitoring, type of blood specimen and adherence challenges, where specific approaches tailored for infants and children may differ from those for adult patients. There are a number of key policy and practice areas for which supportive evidence is limited at this time. Rapid evaluation of initial efforts and experiences scaling up routine VL monitoring for infants and children in LMIC is essential to determine best practices.

## Competing interests

The authors have no competing interests to declare.

## Authors’ contributions

All authors have read and approved the final version.
